# Low Heart Rate Variability in a 2-Minute Electrocardiogram Recording Is Associated with an Increased Risk of Sudden Cardiac Death in the General Population: The Atherosclerosis Risk in Communities Study

**DOI:** 10.1371/journal.pone.0161648

**Published:** 2016-08-23

**Authors:** Ankit Maheshwari, Faye L. Norby, Elsayed Z. Soliman, Selcuk Adabag, Eric A. Whitsel, Alvaro Alonso, Lin Y. Chen

**Affiliations:** 1 Cardiovascular Division, Department of Medicine, University of Minnesota Medical School, Minneapolis, Minnesota, United States of America; 2 Division of Epidemiology and Community Health, School of Public Health, University of Minnesota, Minneapolis, Minnesota, United States of America; 3 Epidemiological Cardiology Research Center (EPICARE), Wake Forest School of Medicine, Winston-Salem, North Carolina, United States of America; 4 Division of Cardiology, Veteran Affairs Medical Center, Minneapolis, Minnesota, United States of America; 5 Departments of Epidemiology and Medicine, University of North Carolina, Chapel Hill, North Carolina, United States of America; 6 Department of Epidemiology, Rollins School of Public Health, Emory University, Atlanta, Georgia, United States of America; University of Minnesota, UNITED STATES

## Abstract

Low heart rate variability (HRV) has been linked to increased total mortality in the general population; however, the relationship between low HRV and sudden cardiac death (SCD) is less well-characterized. The goal of this study was to evaluate the relationship between low HRV and SCD in a community-based cohort. Our cohort consisted of 12,543 participants from the Atherosclerosis Risk in Communities (ARIC) study. HRV measures were derived from 2-minute electrocardiogram recordings obtained during the baseline exam (1987–89). Time domain measurements included the standard deviation of all normal RR intervals (SDNN) and the root mean squared successive difference (r-MSSD). Frequency domain measurements included low frequency power (LF) and high frequency (HF) power. During a median follow-up of 13 years, 215 SCDs were identified from physician adjudication of all coronary heart disease deaths through 2001. In multivariable adjusted Cox proportional hazards models, each standard deviation decrement in SDNN, LF, and HF were associated with 24%, 27% and 16% increase in SCD risk, respectively. Low HRV is independently associated with increased risk of SCD in the general population.

## Introduction

The cardiac autonomic nervous system is comprised of sympathetic and parasympathetic branches. Cardiac function is tightly regulated at multiple levels by the dynamic equilibrium achieved between these systems [1]. Heart rate variability (HRV) is a non-invasive marker for autonomic input to the heart [2]. Increased sympathetic input decreases HRV, whereas increased parasympathetic input increases HRV. Cardiac arrhythmias are often initiated by or occur in patients with enhanced sympathetic and diminished parasympathetic tone [2]. Disturbances in autonomic equilibrium have been implicated in several cardiovascular conditions including myocardial infarction (MI), heart failure, and sudden cardiac death (SCD) [1]. In post-myocardial infarction (MI) patients and heart failure patients, low HRV has been associated with increased risk of all-cause mortality and SCD [3–8]. In the general population, low HRV has been linked to increased risk of cardiovascular events and all-cause mortality [9–11]; however, the relationship between low HRV and SCD risk is less well-characterized. We hypothesized that attenuated HRV is an independent risk factor for SCD in the general population. We tested our hypothesis in the Atherosclerosis Risk in Communities (ARIC) Study—a large community-based cohort study of cardiovascular disease in the United States.

## Methods

### Study Population

The ARIC study is a community-based, prospective cohort study designed to identify and evaluate risk factors, etiology, and clinical manifestations of atherosclerotic coronary heart disease (CHD) in the general population. Between 1987 and 1989, 15,792 men and women aged 45–64 years were recruited and enrolled from four United States communities (Washington County, MD; Forsyth County, NC; Jackson, MS; and suburban Minneapolis, MN). Participants underwent an initial baseline assessment that consisted of a clinical examination, serum laboratory analysis, standard 12-lead electrocardiogram (ECG), 2-minute ECG recording, and a detailed health, behavioral, and socio-demographic history assessment. Follow-up data were obtained from study visits, annual phone calls, local hospital surveillance, and querying of the National Death Index. Approval for the study was obtained from the institutional review board on human research at each participating institution and all participants provided written informed consent. A full list of participating institutional review boards can be found in the supporting information section (S1 Table). Further details outlining study design, outcome ascertainment procedure, and population statistics have been previously described [12].

For our primary analysis, we considered all 15,792 participants at the baseline visit and excluded those with missing covariates (n = 15), missing HRV data (n = 812), HRV recordings of inadequate duration (n = 210), poor quality ECG recordings due to technical issues (n = 636), HRV recordings with inadequate number of acceptable RR intervals (n = 1473), and those who were not white or black from all study sites, and nonwhite from Minneapolis and Washington County (due to small sample size; n = 103) resulting in a final cohort of 12,543 participants. For our secondary analysis, we analyzed a sample of our cohort with lower perceived SCD risk by further excluding those with prevalent CHD or heart failure (n = 1,012) resulting in a final cohort of 11,531 participants.

### Heart Rate Variability Measurement

Measures of HRV were obtained from supine 2-minute ECG recordings obtained during the baseline exam after participants had been resting in the supine position for 20 minutes between 8:30 AM and 11:30 AM. Participants were instructed to refrain from smoking or ingesting caffeine at least 12 hours prior to the procedure. Protocols for data processing, artifact identification, imputation, and quality control of HRV records have been previously described [13,14]. Briefly, the RR interval was converted into beat-to-beat heart rate including a record of the real time of each beat. Potential ectopy and artifact was determined by a computer algorithm that identified all RR intervals outside the upper and lower limits (+/- 25%) of a 5-beat moving average. These beats were removed and the intervening data was linearly smoothened and interpolated using variance preserving imputation software, PREDICT II HRVECG (Arrhythmia Research Technology Inc, Fitchburgh, MA). Records were excluded if >20% of RR intervals were affected to reduce the influence of data imputation. After imputation, heart rate data were converted back to RR intervals for further data processing, time domain analysis, and spectrum analysis using specialized software, PREDICT II HRVECG (Arrhythmia Research and Technology Inc, Fitchburg, MA). In our analysis, time domain measurements included the standard deviation of all normal RR intervals (SDNN) and the root mean squared successive difference (r-MSSD). Frequency domain measurements included low frequency power (LF), defined as the energy in the heart period power spectrum between 0.04 and 0.15 Hz, and high frequency (HF) power, defined as the energy in the heart period power spectrum between 0.15 and 0.40 Hz.

### Sudden Cardiac Death

The outcome of our study was SCD. In ARIC, comprehensive data on all cardiovascular events and deaths was obtained from available death certificates, coroner reports, informant interviews, hospital records, and autopsy reports. The cause of death was adjudicated by the ARIC Morbidity and Mortality Classification Committee following a standard protocol. In order to identify SCD, all fatal CHD events through 2001 in ARIC were reviewed by an independent panel of physicians. Deaths were classified as definite sudden cardiac death, possible sudden cardiac death, not sudden cardiac death, and unclassifiable. Definite sudden cardiac death was defined as a sudden pulseless condition presumed to be of cardiac origin in a previously stable individual without evidence of non-cardiac cause of death. Possible sudden cardiac death was defined as an un-witnessed death in a previously stable (<24 hours) individual without other evidence indicating non-cardiac origin for instantaneous death [15,16]. All deaths classified as SCD had to occur outside of the hospital or in the emergency room. For our analysis, SCD was defined as definite or possible sudden cardiac death.

### Assessment of Other Covariates

The covariates included in our analysis were age, sex, race, study center, cigarette-smoking status, prevalent CHD, heart failure, diabetes (DM), impaired fasting glucose, borderline hypertension (HTN), HTN, beta-adrenergic receptor blocker use, digoxin use, use of anti-arrhythmic drugs (AADs), left ventricular hypertrophy (LVH), and body mass index (BMI). Baseline demographic data, medication use (beta-adrenergic receptor blockers, AADs, and digoxin), and medical history were obtained by ARIC staff from participants during the study visit. AADs include type IA, type IB, type IC and type III anti-arrhythmic medications. Prevalent CHD was defined as a self-reported history of MI, coronary artery bypass grafting, percutaneous coronary intervention, or ECG signs of CHD [17,18]. Heart failure was defined as stage 3 “manifest heart failure” by the Gothenburg criteria or self-reported diagnosis of heart failure [19]. LVH was defined by the Cornell ECG criteria [20,21]. BMI was defined as weight/height^2^ (kg/m^2^) [17]. HTN was defined as systolic blood pressure ≥140 mm Hg, diastolic blood pressure ≥90 mm Hg, or self-reported history of anti-hypertensive medical therapy. Borderline HTN was defined as systolic blood pressure 120–139 mm Hg and/or diastolic blood pressure 80–89 mm Hg. DM was defined as a fasting (minimum of 8 hours) glucose ≥126 gm/dL, non-fasting glucose ≥200 mg/dL, self-reported use of oral hypoglycemic agents or insulin, or self-reported diagnosis of DM [22]. Impaired fasting glucose was defined as fasting (minimum of 8 hours) glucose of 100–125 gm/dL. Smoking status was self-reported. Participants were classified as current smokers and non-current smokers.

### Statistical Analysis

We report means with standard deviations (SDs) for continuous variables and counts with percentages for categorical variables. Person-years at risk were calculated from the date of baseline visit until the date of SCD, other death, loss to follow-up, or end of follow-up, whichever occurred first. The analysis was based on data obtained from 1987–2001.

Initially, we explored the association between HRV measures (as continuous variables) and SCD incidence using restricted cubic splines (knots at the 5th, 27.5th, 50th, 72.5th and 95th percentiles). Frequency domain measures were skewed to the right. Thus, we applied a natural logarithmic transformation to normalize the distribution of HRV frequency domain indices (LF and HF) when used as continuous variables in accordance with the recommendation by the Task Force on HRV research [2]. We used Cox proportional hazards models to estimate the hazard ratios (HRs) and 95% confidence intervals (95% CIs) of HRV measures for SCD. HRV measures were analyzed as tertiles, with the highest tertile as the reference group, and as continuous variables (per 1-SD decrement). We constructed 2 models. Model 1 was adjusted for age, sex, race, and study center. Model 2 was additionally adjusted for BMI, LVH, CHD, heart failure, impaired fasting glucose, DM, beta-blocker use, digoxin use, smoking status (current vs. not current), borderline HTN, HTN, and use of AADs. We conducted a secondary analyses applying Model 2 (without adjustment for CHD or heart failure) to our cohort of participants without prevalent CHD or heart failure. Finally, we performed sex- and race-stratified analyses.

The proportional hazards assumption was assessed with scaled Schoenfeld residuals for both graphical and numerical tests, time interaction terms, and inspection of log negative log survival curves. Modeling assumptions were not violated in any model. Statistical analysis of ARIC data was performed using SAS version 9.2 (SAS Institute Inc., Cary, NC) and STATA 13.0 (StataCorp LP, College Station, TX). All *P* values reported were 2-sided, and statistical significance threshold was chosen as 5%.

## Results

### Study Population

In our cohort of 12,543 participants, we identified 215 SCD events over a median follow-up period of 13 years. This corresponded to an incidence rate (95% confidence interval) of 1.37 (1.19–1.56) per 1000 person-years. Baseline characteristics of our study cohort are shown in Table 1. Participants with SCD were more likely to be male, black and have a history of CHD, DM, HTN, heart failure, or LVH than those without SCD.

**Table 1:** Baseline Participant Characteristics by Sudden Cardiac Death Status, Atherosclerosis Risk in Communities Study, 1987–2001.

Characteristic†	No SCD (n = 12,328)	SCD (n = 215)	P-Value
Age, mean (SD), years	54.0 (5.7)	56.3 (5.7)	<0.0001
Female	7,045 (57.2)	77 (35.8)	<0.0001
Black race	3,173 (25.7)	91 (42.3)	<0.0001
Current smoker	3,135 (25.4)	90 (41.9)	<0.0001
Body Mass Index, mean (SD) kg/m^2^	27.6 (5.3)	28.7 (5.6)	0.003
Diabetes	1,373 (11.1)	75 (34.9)	<0.0001
Hypertension	4,154 (33.7)	136 (63.3)	<0.0001
Heart Failure	527 (4.3)	25 (11.6)	<0.0001
Coronary Heart Disease	498 (4.0)	67 (31.2)	<0.0001
Left Ventricular Hypertrophy	256 (2.1)	17 (7.9)	<0.0001
Use of Digoxin	163 (1.3)	14 (6.5)	<0.0001
Use of Beta-blockers	1268 (10.3)	43 (20.0)	<0.0001
Use of Anti-arrhythmics	86 (0.7)	4 (1.9)	0.07
Heart Rate, mean (SD) ‡	67.7 (10.3)	70.3 (13.8)	.008
SDNN, mean (SD) ms	37.2 (19.7)	31.9 (20.6)	0.002
r-MSSD, mean (SD) ms	29.2 (23.3)	27.3 (28.3)	0.25
Ln LF power, mean (SD) ms^2^	2.7(1.4)	2.0 (1.6)	<0.0001
Ln HF power, mean (SD) ms^2^	2.1 (1.3)	1.6 (1.5)	<0.0001

^†^ Data are presented as no. (%) unless as otherwise stated

^‡^ Heart rate in beats per minute obtained from 2-minute resting ECG obtained during study visit 1

Abbreviations: High Frequency (HF), Low Frequency (LF), Root Mean Squared Successive Difference (r-MSSD), Standard Deviation of Normal RR Intervals (SDNN), Sudden Cardiac Death (SCD).

### HRV and SCD

**Fig 1:**
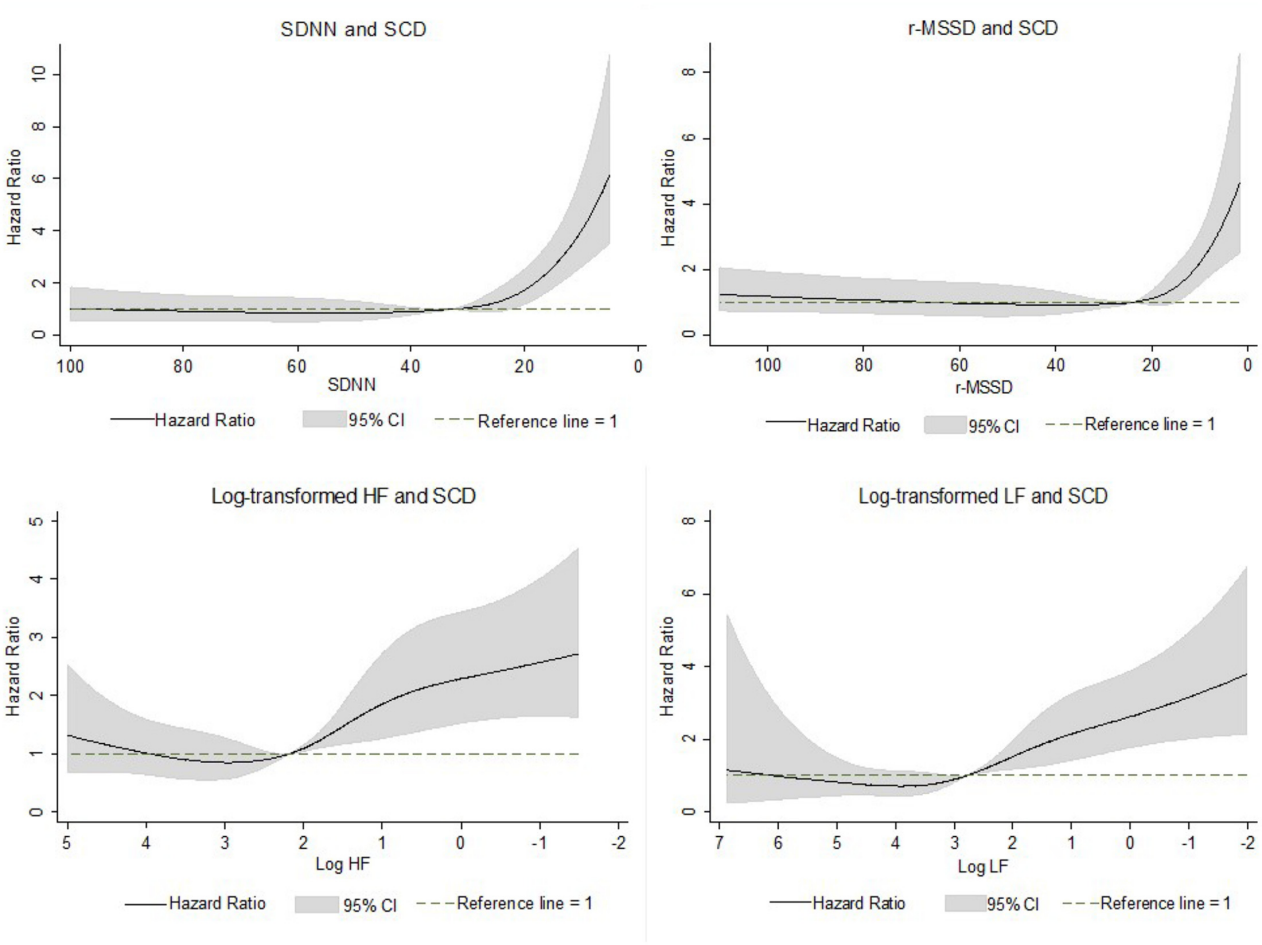
Heart Rate Variability Measures and Sudden Cardiac Death Risk. Association between heart rate variability measures and sudden cardiac death incidence presented as hazard ratios (solid line) and 95% confidence intervals (shaded area). Results from Cox proportional hazards model with heart rate variability measures modeled using restricted cubic splines, adjusted for age, sex, and race. Median value of heart rate variability measures was considered the reference (HR = 1). The x-axis is presented using an inverted scale.

Fig 1 shows the association between HRV measures and SCD risk, modeled as restricted cubic splines. For HRV values below the median, lower HRV measures were associated with higher risk of SCD after adjustment for age, sex, and race. For HRV values above the median, there was no significant relationship between HRV and risk of SCD. Table 2 shows the association between HRV tertiles and the risk of SCD. In the demographically adjusted model (Model 1), compared with the highest tertile, the lowest tertile of SDNN and r-MSSD was significantly associated with an increased risk of SCD. This association was attenuated but remained significant after further adjustment for SCD risk factors and potential confounders (Model 2). When analyzed as a continuous variable in Model 2, each SD decrement in SDNN was associated with a significant 24% increase in SCD risk. r-MSSD was not significantly associated with SCD when analyzed in Model 2 as a continuous variable.

**Table 2:** Hazard Ratios of Sudden Cardiac Death for Heart Rate Variability Measures, Atherosclerosis Risk in Communities Study, 1987–2001.

	Tertile 1	Tertile 2	Tertile 3	P for trend ¶	Per 1-SD decrease #	P-value
SDNN, ms	<27	27–40	>40			
SCD Cases	113	53	49			
Person Years	49,228	56,275	51,559			
SCD Incidence †	2.30 (1.90–2.75)	0.94 (0.71–1.22)	0.95 (0.71–1.25)			
Model 1, HR (95% CI) ‡	2.48 (1.77–3.49)	1.03 (0.70–1.52)	1 (ref)	<0.0001	1.48 (1.24–1.77)	<0.0001
Model 2, HR (95% CI) §	1.80 (1.27–2.55)	0.94 (0.63–1.38)	1 (ref)	0.0002	1.24 (1.06–1.46)	0.009
R-MSSD, ms	<18	18–29	>29			
SCD Cases	96	63	56			
Person Years	48,000	54,672	54,390			
SCD Incidence†	2.00 (1.63–2.43)	1.15 (0.89–1.46)	1.03 (0.79–1.33)			
Model 1, HR (95% CI) ‡	1.98 (1.41–2.78)	1.16 (0.81–1.67)	1 (ref)	<0.0001	1.13 (0.96–1.33)	0.13
Model 2, HR (95% CI) §	1.65 (1.16–2.33)	1.12 (0.78–1.61)	1 (ref)	0.004	1.06 (0.92–1.22)	0.43
LF power, ms^2^	<9	9–25	>25			
SCD Cases	113	58	44			
Person Years	49,367	51,675	56,020			
SCD Incidence†	2.29 (1.90–2.74)	1.12 (0.86–1.44)	0.79 (0.58–1.04)			
Model 1, HR (95% CI) ‡	2.76 (1.94–3.93)	1.43 (0.96–2.12)	1 (ref)	<0.0001	1.51 (1.34–1.69)	<0.0001
Model 2, HR (95% CI) §	1.80 (1.25–2.59)	1.26 (0.85–1.87)	1 (ref)	0.001	1.27 (1.12–1.43)	0.0002
HF power, ms^2^	<5.0	5.0–13.4	>13.4			
SCD Cases	112	51	52			
Person Years	52,223	49,018	55,822			
SCD Incidence†	2.14 (1.77–2.57)	1.04 (0.78–1.36)	0.93 (0.70–1.21)			
Model 1, HR (95% CI) ‡	2.07 (1.47–2.90)	1.09 (0.74–1.61)	1 (ref)	<0.0001	1.35 (1.19–1.53)	<0.0001
Model 2, HR (95% CI) §	1.58 (1.12–2.23)	1.06 (0.72–1.57)	1 (ref)	0.006	1.16 (1.02–1.32)	0.02

^†^ Incidence is reported per 1000 person-years

^‡^ Cox proportional hazard model adjusted for age, sex, race, and study center

^§^ Cox proportional hazard model adjusted for age, sex, race, study center, smoking status (current vs. not current), body mass index, ECG-based left ventricular hypertrophy, hypertension, borderline hypertension, diabetes, impaired fasting glucose, coronary heart disease, heart failure, use of β-blockers, use of digoxin, use of anti-arrhythmic drugs

^¶^ P for trend calculated using the term for tertile categories

^#^ per 1-SD decrease in log-transformed HF and LF for frequency domain

Abbreviations: Confidence Interval (CI), Hazard Ratio (HR), High Frequency (HF), Low Frequency (LF), Root Mean Squared Successive Difference (r-MSSD), Sudden Cardiac Death (SCD), Standard Deviation (SD), Standard Deviation of Normal RR Intervals (SDNN)

In the demographically adjusted model (Model 1), compared with the highest tertile, the lowest tertile of LF and HF spectral power was significantly associated with an increased risk of SCD. This association was attenuated but remained significant after further adjustment in Model 2. When analyzed as a continuous variable in Model 2, each SD decrement in LF and HF was significantly associated with 27% and 16% increase in SCD risk, respectively.

In our sample of participants without prevalent CHD or heart failure, compared with the highest tertiles, the lowest tertiles of LF spectral power, HF spectral power, and SDNN were significantly associated with an increased risk of SCD. When analyzed as a continuous variable, each SD decrement in SDNN and LF spectral power were associated with 25% and 24% increase in SCD risk, respectively (Table 3).

**Table 3:** Hazard Ratios of Sudden Cardiac Death for Heart Rate Variability Measures in Low Risk Cohort (excluding participants with CHD or Heart Failure), Atherosclerosis Risk in Communities Study, 1987–2001.

	Tertile 1	Tertile 2	Tertile 3	P for Trend †	Per 1-SD decrease ‡	P-value
SDNN, ms	<27	27–40	>40			
# Deaths	68	37	36			
HR (95% CI) *	1.63 (1.07–2.47)	0.89 (0.56–1.41)	1 (Ref)	0.01	1.25 (1.02–1.53)	0.03
R-MSSD, ms	<18	18–29	>29			
# Deaths	58	39	44			
HR (95% CI) *	1.34 (0.89–2.03)	0.92 (0.59–1.42)	1 (Ref)	0.15	1.05 (0.88–1.26)	0.57
LF power, ms^2^	<9	9–25	>25			
# Deaths	67	44	30			
HR (95% CI) *	1.97 (1.27–3.06)	1.49 (0.93–2.37)	1 (Ref)	0.002	1.24 (1.06–1.44)	0.007
HF power, ms^2^	<5	5–13.4	>13.4			
# Deaths	65	36	40			
HR (95% CI) *	1.51 (1.00–2.26)	1.03 (0.65–1.62)	1 (Ref)	0.04	1.12 (0.96–1.31)	0.16

* Cox Proportional Hazard Models adjusted for age, sex, race, study center, smoking status (current vs. not current), body mass index, ECG-based left ventricular hypertrophy, hypertension, borderline hypertension, diabetes, impaired fasting glucose, use of β-blockers, use of digoxin, use of anti-arrhythmic drugs

^†^ P for trend calculated using the term for tertile categories

^‡^ per 1-SD decrease in log-transformed HF and LF for frequency domain

Abbreviations: Standard deviation of normal RR intervals (SDNN), root mean squared successive difference (r-MSSD), high frequency (HF), low frequency (LF), Sudden cardiac death (SCD), hazard ration (HR), confidence interval (CI), standard deviation (SD)

We evaluated interactions with age and sex and did not find any significant sex- or race- based interactions with respect to SCD risk (S2 Table). The association between lower HRV and higher risk of SCD was consistently observed in women and men (S3 Table), and blacks and whites (S4 Table).

## Discussion

In this large biracial community-based cohort of middle-aged individuals, we found that lower HRV measured by time-domain and spectral analysis from a resting 2-minute ECG recording was independently associated with higher risk of SCD. This relationship was observed for HRV values below the median, and not above the median, suggesting a threshold effect. From race- and sex-stratified analysis, we found that this relationship was similar in whites and blacks and men and women.

The most prominent clinical risk factors for SCD are CHD and heart failure. Low HRV has been shown to be independently predictive of increased mortality in post-MI patients, heart failure patients, and in cohorts representative of the general population [3,6,9,11,23–25]. It has been independently associated with SCD in small studies conducted in highly selected patients with heart failure [3,7,8,23–25] and MI [4–6,26]. The relationship between HRV and SCD has received limited evaluation in low risk cohorts. Algra et al demonstrated that low HRV was associated with SCD in a selected cohort of predominantly ambulatory patients (75%) with indications for 24-hour ECG monitoring including evaluation for symptoms potentially related to cardiac arrhythmia, post-MI monitoring, and evaluation of anti-arrhythmic therapy [27]. This study, therefore, was not based on unselected subjects from the general population. Further, it was a nested case-control analysis with limited adjustment for SCD risk factors and potential confounders. Stein et al demonstrated that decreased normalized low frequency power, but not time domain HRV, derived from 24-hour ECG recordings was associated with SCD in a subset of participants from the Cardiovascular Health Study using a nested case-control analysis [28]. We extend current knowledge by demonstrating for the first time that low HRV derived from a 2-minute ECG recording is prospectively associated with an increased risk of SCD independent of established cardiovascular risk factors in a low-risk, middle-aged, community-based setting, even after excluding participants with CHD or heart failure.

We considered several mechanisms to explain this association. The majority of SCDs result from unstable ventricular tachyarrhythmias (VTAs) occurring in community-dwelling individuals with undetected but significant CHD [29–35]. Such arrhythmias can be mediated by increased sympathethic and/or decreased parasympathetic input to the heart, which may be detected by HRV analysis. In canine models, VTAs have been provoked by increased sympathetic activity, especially in the setting of mechanical coronary occlusion, while vagal stimulation has been shown to increase the VTA threshold [36–39]. Adverse autonomic remodeling evidenced by sympathetic nerve sprouting and hyper-innervation has been linked to the development of VTAs in canine models and observed in the myocardium of patients with a history of VTAs [39].

Autonomic imbalance, however, is a non-specific marker linked with several cardiovascular diseases. Low HRV, in turn, has been independently associated with many SCD risk factors including CHD [9] and obstructive CHD [40]. While we adjusted for cardiovascular risk factors in our models, we were unable to account for unreported or undetected disease. Thus, the relationship between low HRV and increased SCD risk may also be explained by underlying risk factors such as subclinical CHD.

The current paradigm for non-invasive SCD risk stratification in the general population is limited. With the majority of SCDs occurring as the first manifestation of undetected, untreated CHD, the discovery of novel SCD predictors capable of identifying at-risk individuals in the general population earlier in the natural history of disease is paramount. Several such parameters have been investigated including proteomic, electrophysiological, genetic, structural, and autonomic variables [41,42]. While independent relationships with increased SCD risk have been identified, large-scale investigations for purposes of risk stratification in the general population are limited. Our findings suggest that low HRV in a 2-minute ECG is an independent risk factor for SCD. The population attributable risk, however, is modest and the clinical utility, if any, of our findings is not established and will need to be clarified.

The principal strengths of our study include the prospective design, length of follow up (>10 years), large cohort size (>10,000 participants), and the inclusion of whites and blacks. Some limitations should be noted. First, HRV data were only obtained from 2-minute ECG recordings. Short (2–15 min) recordings may not encapsulate effects of the circadian rhythm and daily activity compared with long (24 hour) recordings. However, HRV measures derived from short and even ultra-short (10s) recordings are reliable [43–45]. As such, short recordings have been employed in numerous epidemiological analyses as they pose a significant advantage over long recordings with respect to feasibility. Second, approximately 20% of ARIC participants were excluded due to missing HRV data, technical issues with 2-minute ECG recordings, and artifact. The missing data and technical issues resulted from logistical problems that were likely random in nature; thus, it would not have biased our results. In the HRV estimation protocol, potential ectopy and artifact was identified by statistical criteria imposed on RR intervals, not morphological analysis of the QRS complex. Thus, we cannot be certain that all QRS complexes analyzed indeed resulted from sinus node depolarization.

In summary, low HRV in a 2-minute resting ECG is independently associated with an increased risk of SCD in the general population. Further studies are needed to determine if the inclusion of HRV in a multi-marker approach would improve risk prediction of SCD in the general population.

## Supporting Information

S1 Table List of participating ARIC centers. (DOCX)

S2 Table Sex and Race Interactions. (DOCX)

S3 Table Sex-Stratified Hazard Ratios of Sudden Cardiac Death for Heart Rate Variability Measures, Atherosclerosis Risk in Communities Study, 1987–2001. (DOCX)

S4 Table Race-Stratified Hazard Ratios of Sudden Cardiac Death for Heart Rate Variability Measures, Atherosclerosis Risk in Communities Study, 1987–2001. (DOCX)

## Abbreviations

AADs: Anti-Arrhythmic Drugs
ARIC: Atherosclerosis Risk in Communities
BMI: Body Mass Index
CHD: Coronary Heart Disease
DM: Diabetes Mellitus
ECG: Electrocardiogram
HF: High Frequency Power
HR: Hazard Ratio
HRV: Heart Rate Variability
HTN: Hypertension
LF: Low Frequency Power
LVH: Left Ventricular Hypertrophy
MI: Myocardial Infarction
r-MSSD: Root Mean Squared Successive Difference
SCD: Sudden Cardiac Death
SD: Standard Deviation
SDNN: Standard Deviation of All Normal RR Intervals
VTA: Ventricular Tachyarrhythmia
