# Supplementary material for: Low Heart Rate Variability in a 2-Minute Electrocardiogram Recording Is Associated with an Increased Risk of Sudden Cardiac Death in the General Population: The Atherosclerosis Risk in Communities Study
Source: PLoS One. 2016 Aug 23;11(8):e0161648. doi: 10.1371/journal.pone.0161648 (PMC4995012; doi:10.1371/journal.pone.0161648)
Supplement: S1 Table — (DOCX) [file pone.0161648.s001.docx]

| National Heart, Lung, and Blood Institute |
| --- |
| University of North Carolina at Chapel Hill, NC |
| Forsyth County Field Center – Wake Forest Baptist Medical Center, Winston-Salem, NC |
| Jackson Field Center - University of Mississippi Medical Center, Jackson, MS |
| Minneapolis Suburbs Field Center – University of Minnesota, Minneapolis, MN |
| Washington County Field Center - Johns Hopkins University, Baltimore, MD |
| Echocardiogram Reading Center - Brigham and Women's Hospital, Boston, MA |
| Atherosclerosis Laboratory - Baylor College of Medicine, Houston, TX |
| Genetics Laboratory - University of Texas Health Science Center, Houston, TX |
| DLCO Reading Center - National Institute of Environmental Health Sciences, Research Triangle Park, NC |
| ECG Reading Center - EPICARE- Wake Forest University Health Sciences, Winston-Salem, NC |
| Pulmonary Function Reading Center – Athens, GA |
| Pulse Wave Velocity Reading Center - University of Texas, Austin, TX |
| ECG Reading Center: Surveillance - University of Minnesota, Minneapolis, MN |
| Clinical Chemistry Laboratory - University of Minnesota, Minneapolis, MN |
| MRI Reading Center - Mayo Clinic, Rochester, MN |
| MRI Vascular Reading Center - Johns Hopkins Hospital, Baltimore, MD |
| Retinal Reading Center - University of Wisconsin, Madison, WI |
