# Supplementary material for: Low Heart Rate Variability in a 2-Minute Electrocardiogram Recording Is Associated with an Increased Risk of Sudden Cardiac Death in the General Population: The Atherosclerosis Risk in Communities Study
Source: PLoS One. 2016 Aug 23;11(8):e0161648. doi: 10.1371/journal.pone.0161648 (PMC4995012; doi:10.1371/journal.pone.0161648)
Supplement: S2 Table — (DOCX) [file pone.0161648.s002.docx]

| HRV measure | P for sex interaction† | P for race interaction† |
| --- | --- | --- |
| SDNN | 0.72 | 0.21 |
| r-MSSD | 0.59 | 0.32 |
| LF power | 0.47 | 0.65 |
| HF power | 0.81 | 0.74 |

† Cox Proportional Hazard Models adjusted for age, sex, race, study center, smoking status (current vs. not current), body mass index, ECG-based left ventricular hypertrophy, hypertension, borderline hypertension, diabetes, impaired fasting glucose, coronary heart disease, heart failure, use of β-blockers, use of digoxin, use of anti-arrhythmic drugs

Abbreviations: Heart Rate Variability (HRV), High Frequency (HF), Low Frequency (LF), Root Mean Squared Successive Difference (r-MSSD), Sudden Cardiac Death (SCD), Standard Deviation of Normal RR Intervals (SDNN)
