# Supplementary material for: Low Heart Rate Variability in a 2-Minute Electrocardiogram Recording Is Associated with an Increased Risk of Sudden Cardiac Death in the General Population: The Atherosclerosis Risk in Communities Study
Source: PLoS One. 2016 Aug 23;11(8):e0161648. doi: 10.1371/journal.pone.0161648 (PMC4995012; doi:10.1371/journal.pone.0161648)
Supplement: S3 Table — (DOCX) [file pone.0161648.s003.docx]

|  | Tertile 1 | Tertile 2 | Tertile 3 | P for trend ‡ | Per 1-SD decrease § | P-value |
| --- | --- | --- | --- | --- | --- | --- |
| SDNN, ms | <27 | 27-40 | >40 |  |  |  |
| Female† | 1.47 (0.81-2.68) | 1.02 (0.52-1.98) | 1 (ref) | 0.15 | 1.26 (0.95-1.68) | 0.11 |
| Male† | 2.06 (1.34-3.18) | 0.91 (0.56-1.49) | 1 (ref) | 0.0003 | 1.25 (1.03-1.53) | 0.03 |
| R-MSSD, ms | <18 | 18-29 | >29 |  |  |  |
| Female† | 1.24 (0.72-2.13) | 0.81 (0.44-1.48) | 1 (ref) | 0.45 | 1.03 (0.81-1.32) | 0.80 |
| Male† | 2.07 (1.31-3.29) | 1.41 (0.88-2.27) | 1 (ref) | 0.002 | 1.08 (0.90-1.30) | 0.40 |
| LF power, ms^2^ | <9 | 9-25 | >25 |  |  |  |
| Female† | 1.84 (0.95-3.58) | 1.84 (0.92-3.70) | 1 (ref) | 0.10 | 1.29 (1.05-1.58) | 0.02 |
| Male† | 1.87 (1.21-2.90) | 1.04 (0.64-1.70) | 1 (ref) | 0.003 | 1.27 (1.09-1.49) | 0.003 |
| HF power, ms^2^ | <5.0 | 5.0-13.4 | >13.4 |  |  |  |
| Female† | 1.61 (0.92-2.80) | 1.03 (0.56-1.91) | 1 (ref) | 0.08 | 1.21 (0.97-1.50) | 0.09 |
| Male† | 1.56 (1.01-2.43) | 1.04 (0.63-1.73) | 1 (ref) | 0.03 | 1.16 (0.99-1.36) | 0.07 |

† Cox Proportional Hazard Models adjusted for age, sex, race, study center, smoking status (current vs. not current), body mass index, ECG-based left ventricular hypertrophy, hypertension, borderline hypertension, diabetes, impaired fasting glucose, coronary heart disease, heart failure, use of β-blockers, use of digoxin, use of anti-arrhythmic drugs

‡ P for trend calculated using the term for tertile categories

§ per 1-SD decrease in log-transformed HF and LF for frequency domain

Abbreviations: Confidence Interval (CI), Hazard Ratio (HR), High Frequency (HF), Low Frequency (LF), Root Mean Squared Successive Difference (r-MSSD), Sudden Cardiac Death (SCD), Standard Deviation (SD), Standard Deviation of Normal RR Intervals (SDNN), Sudden Cardiac Death (SCD)
