# Supplementary material for: Low Heart Rate Variability in a 2-Minute Electrocardiogram Recording Is Associated with an Increased Risk of Sudden Cardiac Death in the General Population: The Atherosclerosis Risk in Communities Study
Source: PLoS One. 2016 Aug 23;11(8):e0161648. doi: 10.1371/journal.pone.0161648 (PMC4995012; doi:10.1371/journal.pone.0161648)
Supplement: S4 Table — (DOCX) [file pone.0161648.s004.docx]

|  | Tertile 1 | Tertile 2 | Tertile 3 | P for trend ‡ | Per 1-SD decrease § | P-value |
| --- | --- | --- | --- | --- | --- | --- |
| SDNN, ms | <27 | 27-40 | >40 |  |  |  |
| Black† | 2.46 (1.40-4.32) | 1.11 (0.59-2.11) | 1 (ref) | 0.03 | 1.46 (1.12-1.89) | 0.005 |
| White† | 1.57 (1.00-2.47) | 0.89 (0.54-1.47) | 1 (ref) | 0.0005 | 1.14 (0.93-1.40) | 0.22 |
| R-MSSD, ms | <18 | 18-29 | >29 |  |  |  |
| Black† | 2.16 (1.29-3.60) | 1.27 (0.74-2.18) | 1 (ref) | 0.12 | 1.22 (0.95-1.56) | 0.12 |
| White† | 1.41 (0.88-2.25) | 1.03 (0.62-1.69) | 1 (ref) | 0.003 | 1.00 (0.85-1.18) | 0.99 |
| LF power, ms^2^ | <9 | 9-25 | >25 |  |  |  |
| Black† | 2.14 (1.18-3.86) | 1.64 (0.86-3.13) | 1 (ref) | 0.04 | 1.24 (1.03-1.49) | 0.02 |
| White† | 1.58 (0.99-2.53) | 1.03 (0.62-1.70) | 1 (ref) | 0.01 | 1.28 (1.08-1.52) | 0.004 |
| HF power, ms^2^ | <5.0 | 5.0-13.4 | >13.4 |  |  |  |
| Black† | 1.77 (1.07-2.93) | 1.07 (0.60-1.88) | 1 (ref) | 0.08 | 1.24 (1.03-1.49) | 0.03 |
| White† | 1.49 (0.93-2.39) | 1.11 (0.65-1.90) | 1 (ref) | 0.02 | 1.11 (0.94-1.33) | 0.22 |

† Cox Proportional Hazard Models adjusted for age, sex, race, study center, smoking status (current vs. not current), body mass index, ECG-based left ventricular hypertrophy, hypertension, borderline hypertension, diabetes, impaired fasting glucose, coronary heart disease, heart failure, use of β-blockers, use of digoxin, use of anti-arrhythmic drugs

‡ P for trend calculated using the term for tertile categories

§ per 1-SD decrease in log-transformed HF and LF for frequency domain

Abbreviations: Confidence Interval (CI), Hazard Ratio (HR), High Frequency (HF), Low Frequency (LF), Root Mean Squared Successive Difference (r-MSSD), Sudden Cardiac Death (SCD), Standard Deviation (SD), Standard Deviation of Normal RR Intervals (SDNN), Sudden Cardiac Death (SCD)
